# Paxillin-dependent regulation of *IGF2* and *H19* gene cluster expression

**DOI:** 10.1242/jcs.170985

**Published:** 2015-08-15

**Authors:** Pavel Marášek, Rastislav Dzijak, Irina Studenyak, Jindřiška Fišerová, Lívia Uličná, Petr Novák, Pavel Hozák

**Affiliations:** 1 Department of Biology of the Cell Nucleus, Institute of Molecular Genetics AS CR, Prague 142 20, Czech Republic; 2 Faculty of Science, Charles University in Prague, Prague 128 43, Czech Republic; 3 Department of Genome Integrity, Institute of Molecular Genetics AS CR, Prague 142 20, Czech Republic; 4 Laboratory of Structural Biology and Cell Signaling, Institute of Microbiology AS CR, Prague 142 00, Czech Republic

**Keywords:** Paxillin, Cohesin, Imprinting, *IGF2*, *H19*, Enhancer

## Abstract

Paxillin (PXN) is a focal adhesion protein that has been implicated in signal transduction from the extracellular matrix. Recently, it has been shown to shuttle between the cytoplasm and the nucleus. When inside the nucleus, paxillin promotes cell proliferation. Here, we introduce paxillin as a transcriptional regulator of *IGF2* and *H19* genes. It does not affect the allelic expression of the two genes; rather, it regulates long-range chromosomal interactions between the *IGF2* or *H19* promoter and a shared distal enhancer on an active allele. Specifically, paxillin stimulates the interaction between the enhancer and the *IGF2* promoter, thus activating *IGF2* gene transcription, whereas it restrains the interaction between the enhancer and the *H19* promoter, downregulating the *H19* gene. We found that paxillin interacts with cohesin and the mediator complex, which have been shown to mediate long-range chromosomal looping. We propose that these interactions occur at the *IGF2* and *H19* gene cluster and are involved in the formation of loops between the *IGF2* and *H19* promoters and the enhancer, and thus the expression of the corresponding genes. These observations contribute to a mechanistic explanation of the role of paxillin in proliferation and fetal development.

## Abstract (precis)

**Summary:** A new regulatory pathway whereby paxillin mediates the expression of two growth-related genes, *IGF2* and *H19*, is presented, uncovering a role for paxillin in cell proliferation.

## INTRODUCTION

Multicellular organisms are composed of many different cell types that become organized into distinct tissues and organs during development. Focal adhesions are among the key structures required for these cellular rearrangements, because they mediate the transfer of information about the cellular environment and are essential components of the response mechanism (Zamir and Geiger, 2001). A number of proteins are involved in focal adhesion regulation. Interestingly, focal adhesion proteins can be found not only at focal adhesion contacts but also inside the nucleus and they can shuttle out of it and back in (Hervy et al., 2006). This allows them to participate in a crosstalk between the nucleus and the cytoplasm by converting the extracellular signals into altered gene expression. Given that focal adhesion proteins play an important role in crucial cellular processes, including cell proliferation, survival and differentiation (reviewed in Hervy et al., 2006), their occurrence in the nucleus suggests that they are involved in a new mechanism for regulation of these processes. However, nuclear functions of adhesion proteins are just beginning to be understood.

The adaptor protein paxillin is one of the major components of focal adhesions. Its principal function is a coordination and propagation of downstream signaling from transmembrane integrin and growth factor receptors (reviewed in Brown and Turner, 2004). Paxillin has also been shown to shuttle between focal adhesions and the nucleus. The block of CRM1-dependent export pathway causes accumulation of paxillin inside the nucleus (Woods et al., 2002). The shuttling is regulated by phosphorylation and dephosphorylation because nuclear localization of paxillin requires mitogen-activated protein kinase (MAPK)-dependent phosphorylation (Sen et al., 2012), whereas its export from the nucleus requires dephosphorylation (Dong et al., 2009).

Paxillin has been recently linked to the regulation of *H19* gene. Overexpression of paxillin downregulates the expression of *H19* in mouse 3T3 cells and directly suppresses the mouse *H19* promoter (Dong et al., 2009). This gene produces a 2.3-kb long, capped, spliced and polyadenylated non-coding RNA (Brannan et al., 1990; Milligan et al., 2002). The first exon of *H19* RNA encodes two conserved microRNAs (miRNAs), miR-675-3p and miR-675-5p, that are proposed to be responsible for proliferation-repressive function of *H19* (Mineno et al., 2006; Cai and Cullen, 2007; Keniry et al., 2012). The *H19* and insulin-like growth factor (*IGF2*) gene form a reciprocally imprinted cluster (*IGF2*/*H19*) located on the human chromosome 11 (Reik and Walter, 2001). Imprinting restricts the expression of these genes to only a single allele*. H19* expression is restricted to the maternal allele, whereas *IGF2* is transcribed only from the paternal one (reviewed in Bartolomei and Ferguson-Smith, 2011). In addition, paternal expression of *IGF2* and maternal expression of *H19* are mechanistically coupled (Ratajczak, 2012). The current model of the imprinting mechanism includes an imprinting control region (ICR) positioned between the two genes, an enhancer located downstream of both of them, and long-range chromosomal interactions orchestrated by a cohesin complex and a CCCTC-binding factor (CTCF; reviewed in MacDonald, 2012). The zinc-finger insulator protein CTCF binds to the maternal unmethylated ICR and blocks the access of the enhancer to the *IGF2* promoter (Bell and Felsenfeld, 2000; Hark et al., 2000). Paternal methylation of the *H19* ICR inhibits CTCF binding, thus allowing the enhancer to activate the *IGF2* promoter on the paternal chromosome (Murrell et al., 2004; Kurukuti et al., 2006). Maintaining this imprinting pattern is crucial for cell growth and development (reviewed in Ishida and Moore, 2013).

The transcription of the *IGF2*/*H19* locus is further controlled by an evolutionarily conserved cohesin complex (Parelho et al., 2008; Wendt et al., 2008; Nativio et al., 2009) composed of four core subunits, SMC1A, SMC3, SCC1 (also known as RAD21) and SCC3 (also known as SA2 and STAG2) (Guacci et al., 1997; Michaelis et al., 1997; Losada et al., 1998). These proteins assemble in a ring-like structure (Haering et al., 2002), topologically entrapping DNA strands as a ring (Haering et al., 2002; Gruber et al., 2003). Cohesin (along with CTCF) regulates higher order chromatin conformation at the *IGF2*/*H19* locus, forming distinct intrachromosomal loops (Nativio et al., 2009; reviewed in MacDonald, 2012). In addition, cohesin along with the protein complex known as mediator of RNA polymerase II (hereafter mediator) has been shown to mediate long-range looping between distal enhancers and the pluripotency-regulated genes (Kagey et al., 2010), which is important for maintenance of their expression (Kagey et al., 2010; Conaway and Conaway, 2011). However, the link between paxillin and *IGF2*/*H19* transcription regulators has remained elusive. Our study expands on the current understanding of the role of paxillin in the expression of *H19* and its functional antagonist *IGF2*, and demonstrates that paxillin regulates long-range chromosomal interactions formed between the promoters of the active *IGF2/H19* alleles and the enhancer, and thus mediates the expression of the *IGF2/H19* gene cluster. Finally, we show that the interaction of paxillin, cohesin and mediator plays a role in this regulation.

## RESULTS

### Paxillin knockdown promotes gene *H19* expression and slows down proliferation in human HepG2 cells

**Fig. 1.**
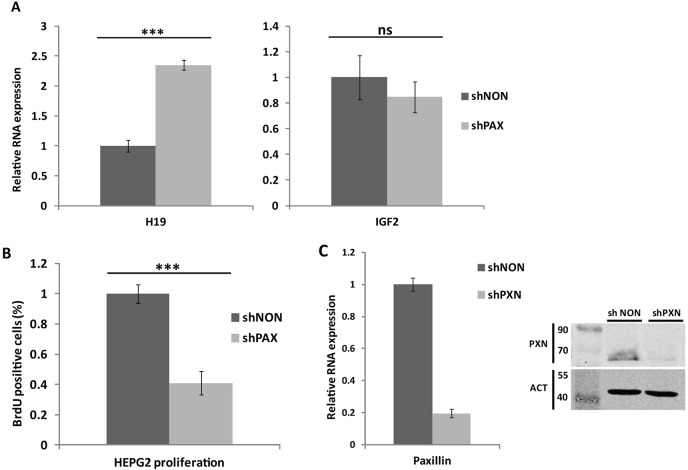
**Paxillin affects the expression of *H19* and regulates cell proliferation in HepG2 cell line.** (A) Quantitative PCR analysis showing that paxillin depletion by shRNA (shPXN) results in upregulation of *H19* compared to control (shNON); no effect on *IGF2* was observed in HepG2 cells. (B) Paxillin depletion (shPXN) resulted in a decreased number of cells incorporating BrdU, that is, undergoing replication. (C) Expression level of paxillin mRNA and protein level of paxillin in paxillin-depleted (shPXN) and control (shNON) HepG2 cells. qPCR data were normalized to the *GAPDH* gene and then to the control shNON sample. Actin (ACT) was used as a control of protein amount. Data are shown as mean±s.d. (*n*≥3). ***P<0.001; ns, *P*>0.05 (Student's *t*-test).

Overexpression of paxillin in mouse cells has been shown to block *H19* expression (Dong et al., 2009). To explore the role of human paxillin in the expression of *H19*, we depleted paxillin from HepG2 cells by lentiviral transduction of short hairpin RNA (shRNA) directed against human paxillin. As expected, paxillin shRNA (shPXN) reduced the level of paxillin transcripts (Fig. 1C) and the protein level (Fig. 1C) to ∼20% of controls. Importantly, in accordance with published data (Dong et al., 2009), the depletion of paxillin resulted in upregulation of *H19* transcription by approximately twofold (Fig. 1A) compared to control cells. Three different clones of shPXN were tested with similar results. The clone with the highest knockdown efficacy was selected for further experiments.

Genes *H19* and *IGF2* form a gene cluster on human chromosome 11 and share DNA as well as protein regulatory elements (Wendt et al., 2008; Yao et al., 2010). Therefore, we also tested the effect of paxillin depletion on *IGF2* expression in HepG2 cells. We detected a slight decrease in *IGF2* expression, but the difference was insignificant (*P*>0.05, Fig. 1A).

*H19* and *IGF2* genes have the opposite effect on cell proliferation; *IGF2* encodes a growth-promoting peptide hormone, whereas the *H19* non-coding RNA gives rise to proliferation-repressing miRNAs (Cai and Cullen, 2007; Gabory et al., 2010; Dey et al., 2014). We thus wondered how paxillin depletion in HepG2 cells would affect cell growth, and whether differences in cell growth would reflect the expression changes of proliferation-regulatory genes. Proliferating cells were identified by incubation with the synthetic thymidine analog BrdU, which is incorporated into newly synthesized DNA during S phase. A BrdU assay showed that the knockdown of paxillin and subsequent *H19* upregulation resulted in fewer cells incorporating BrdU within DNA (Fig. 1B), that is, undergoing replication. Thus, consistent with the growth-suppressing function of *H19*, when paxillin level is low the proliferation of HepG2 cells is accordingly slower.

### Paxillin knockdown does not impair imprinting of the *IGF2*/*H19* gene cluster

**Fig. 2.**
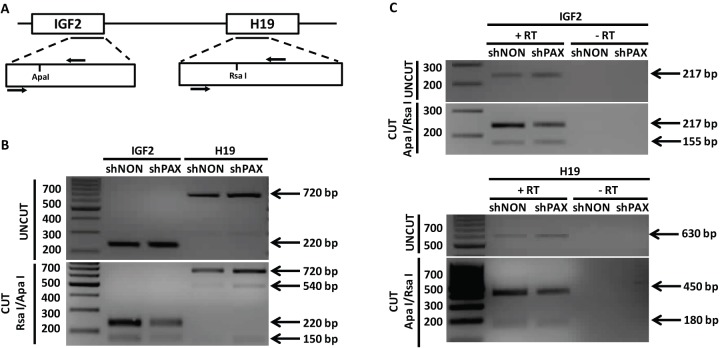
**Paxillin depletion does not change allele-specific expression of *IGF2* and *H19*.** (A) A diagram of *IGF2*/*H19* locus and the amplified regions; arrows show the location of primers for the analyzed polymorphisms (*IGF2*, ApaI; *H19*, RsaI). (B) Analysis of single nucleotide polymorphisms (SNPs) in genomic DNA of SAOS2 cells. Restriction analysis showed heterozygosity of *H19* SNP rs2839704 and *IGF2* SNP rs680 in genomic DNA of SAOS2 cells. *IGF2* SNP rs680 (uncut, 220 bp; cut, 150 and 70 bp; the 70 bp fragment migrated out of the gel) and RsaI for *H19* SNP rs2839704 (uncut, 720 bp, cut, 540 and 180 bp; the 180 bp fragment is not marked). The restriction site is present in one allele only. The *H19* primers are spanning an intron and, therefore, the PCR fragment amplified from genomic DNA is bigger than the cDNA-amplified fragment. (C) *IGF2* showed biallelic expression in paxillin-depleted (shPXN) as well as in control SAOS2 cells (shNON). An undigested 217-bp fragment and a digested 155-bp fragment were present after the restriction digest, showing that both alleles are transcribed. *H19* retained monoallelic expression in both shPXN and shNON SAOS2 cells. There was no undigested 656-bp fragment, and only digested 488-bp and 168-bp fragments were detected after restriction digest, suggesting that only one allele is transcribed. *IGF2* SNP rs680, cut with Apa I (uncut, 220 bp; cut, 150 and 70 bp; the 70 bp fragment migrated out of the gel); *H19* SNP rs2839704, cut with RsaI (uncut, 630 bp; cut, 450 and 180 bp). The restriction site is present in one allele only. +RT, reverse transcription included; −RT, reverse transcription omitted to check for possible genomic DNA contamination.

To investigate whether the change in expression of *H19* following the paxillin knockdown is a consequence of deregulated imprinting, we examined allele-specific expression of *H19* and *IGF2*. To distinguish between the alleles, we employed single nucleotide polymorphism (SNP) assays in SAOS2 cells, where the the *IGF2*/*H19* polymorphisms in genomic DNA are heterozygous (Fig. 2A,B; also see Discussion). RNA isolated from control and paxillin-knockdown cells was reverse transcribed into cDNA, the region covering SNP rs680 for *IGF2* and rs2839704 for *H19* was then amplified by PCR, and digested with ApaI or RsaI for *IGF2* SNP or *H19* SNP, respectively (Fig. 2C). The analysis clearly showed that *H19* was still expressed only from the maternal allele both in control and paxillin-knockdown SAOS2 cells, thus excluding the hypothesis that the lack of paxillin affects imprinting of *H19*. *IGF2* showed biallelic expression in both control and paxillin-knockdown cell lines. Thus, *IGF2* imprinting is deregulated regardless of paxillin expression and independently of *H19*.

The monoallelic expression of *H19* is further controlled by methylation of the ICR lying upstream of *H19*. To test whether paxillin plays a role in ICR methylation, we tracked changes in the ICR methylation pattern after paxillin-knockdown in SAOS2 cells. Isolated genomic DNA was treated with bisulfite, and the region corresponding to the ICR, covering 13 CpG islands (as specified in supplementary material Fig. S1A), was amplified by PCR, cloned into a vector and sequenced. The selected region contained a polymorphic site rs2071094 making it possible to differentiate between alleles (parental origin is unknown). Therefore, we then analyzed the methylation status of each allele. Consistent with the observation that paxillin had no effect on the allelic expression of *IGF2*/*H19* (Fig. 2C), ICR methylation was properly maintained in the control sample as well as in the paxillin-knockdown cells, and no gain of methylation on the second allele was detected (supplementary material Fig. S1B). Thus, we conclude that the ICR imprinting barrier of the *IGF2*/*H19* cluster is unaffected by paxillin knockdown.

### Paxillin regulates *IGF2* and *H19* promoter activity through their shared distant enhancer

**Fig. 3.**
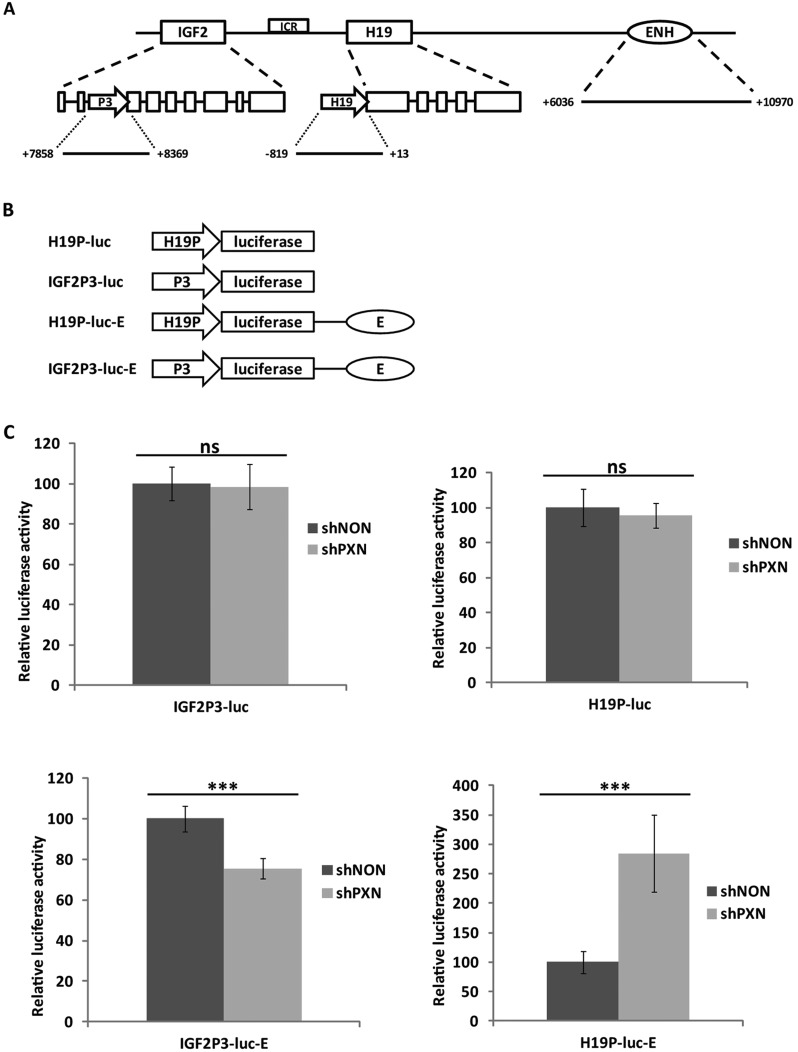
**Paxillin regulates the *IGF2* P3 and *H19* promoters through their shared enhancer.** (A) A scheme showing genomic context of the cloned sections; arrows are promoters, boxes are exons. The *IGF2* P3 corresponds to the sequence +7858 to +8369 bp relative to the *IGF2* exon 1 start. The *H19* promoter represents the sequence −819 to +13 bp relative to the *H19* transcription start site. The enhancer constitutes the sequence +6036 to +10,970 bp relative to the *H19* transcription start site. (B) A scheme of luciferase constructs. *H19*P-luc or *IGF2*P3-luc consists of Firefly luciferase gene (luc) driven by the *H19* promoter (*H19*P) or *IGF2* promoter 3 (*IGF2*P3), respectively. *H19*P-luc-E or *IGF2*P3-luc-E additionally contained an enhancer sequence (E) located downstream of the luciferase gene. (C) Paxillin requires the enhancer to regulate the *IGF2* P3 and *H19* promoters. Depletion of paxillin from HepG2 cells (shPXN) increases the activity of *H19* promoter and decreases the activity of the *IGF2* promoter through their shared enhancer compared to control (shNON). Data were normalized to the *Renilla* luciferase activity. Data are shown as mean±s.d. (*n*≥3). ***P<0.001; ns, *P*>0.05 (Student's *t*-test).

To explore the effect of paxillin on *IGF2* and *H19* promoter activity, we constructed luciferase reporter vectors. *IGF2* and *H19* promoters (Fig. 3A) from HepG2 genomic DNA were inserted upstream of the Firefly luciferase gene (p*H19*P-luc, p*IGF2*P3-luc; Fig. 3B). Whereas *H19* is transcribed from a single promoter, the expression of *IGF2* is controlled by four different promoters (P1, P2, P3, P4; Sussenbach, 1989; Holthuizen et al., 1990; van Dijk et al., 1991). As we found that 95% of *IGF2* RNA was transcribed from P3 in HepG2 cells (data not shown), we used the P3 promoter in further experiments. Then, we examined luciferase activity in paxillin-knockdown as well as in control HepG2 cells. Surprisingly, no change in luciferase activity was detected after paxillin knockdown (Fig. 3C), suggesting that promoter activity was unaffected by reduced paxillin level.

The expression of *IGF2*/*H19* genes is mediated by a distant enhancer located downstream of the *H19*-coding region (Fig. 3A). Both genes, *IGF2* and *H19*, compete for this enhancer (Leighton et al., 1995a; Kaffer et al., 2000). We adopted the construction of the reporter plasmids for testing of enhancer-stimulated expression described by Ishihara et al. (Ishihara et al., 2006). We added a 5-kb-long enhancer region (+6033 to +10,972 relative to *H19* transcription start site) into the luciferase reporter vectors downstream of the luciferase gene, mimicking its position relative to the *IGF2*/*H19* genes (p*H19*P-luc-E, p*IGF2*P3-luc-E; Fig. 3B), and measured luciferase activity in paxillin knockdown as well as control HepG2 cells. Strikingly, paxillin knockdown resulted in an approximately threefold increase in luciferase activity when driven by the *H19* promoter-enhancer, whereas a 0.8-fold decrease was observed when it was driven by the *IGF2* P3 enhancer (Fig. 3C). Interestingly, this result is consistent with the observed expression changes in expression of the respective endogenous genes in paxillin-knockdown cells (see Fig. 1A).

Thus, the lack of paxillin increases the activity of *H19* promoter through its enhancer and decreases the activity of the *IGF2* promoter. In other words, normal paxillin levels are likely to have a suppressive effect on the *H19* promoter and a positive effect on the *IGF2* promoter. Importantly, paxillin does not regulate these promoters directly, but through the distant shared enhancer.

### Paxillin interacts with the *IGF2* P3 and *H19* promoters and their enhancer

**Fig. 4.**
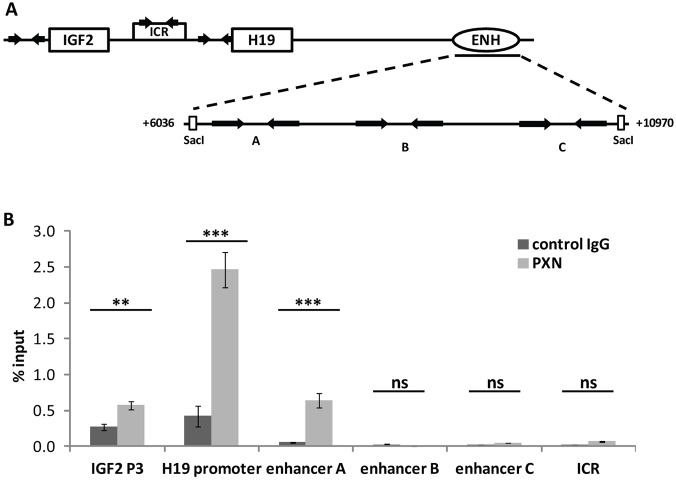
**Paxillin interacts with the *IGF2* P3 and *H19* promoters and their enhancer.** (A) A scheme of *IGF2*/*H19* cluster with arrows indicating the designed primers. (B) ChIP using anti-paxillin antibody (PXN) or mouse IgG (control IgG) showed a specific enrichment of paxillin at the *IGF2* promoter 3 (*IGF2* P3), the *H19* promoter and the 5′ end of the enhancer (enhancer A) in HepG2 cells. No binding to the ICR region was detected. For the enhancer sequence (+6033 to +10,972 bp) we used three different primer sets (A, B and C). Set A amplified the region at the 5′ end (+6189 to +6363) of the enhancer, set B the region approximately in the middle (+7814 to +8038) and set C the region close to the 3′ end (+9978 to +10,217). The GAPDH gene was used as a negative control site. Data are shown as mean±s.d. (*n*≥3). ***P<0.001; **P<0.01 (Student's *t*-test).

Next, we investigated whether the regulation of *IGF2*/*H19* promoters by paxillin occurs through an interaction of paxillin with the regulatory DNA elements of both genes, including the *IGF2* and *H19* promoters, and the shared enhancer or ICR. Therefore, we performed chromatin immunoprecipitation (ChIP) to detect paxillin–DNA complexes in HepG2 cells. ChIP analysis revealed that paxillin mostly interacted with the *H19* promoter. The interaction of paxillin with *IGF2* P3 promoter and the shared enhancer was weaker, and no binding to the ICR region was detected (Fig. 4B). For the enhancer sequence (+6033 to +10,972 bp), we used three different primer sets (A, B and C in Fig. 4A) to cover the whole region. Set A amplified the region at the 5′ end (+6189 to +6363) of the enhancer, set B the region approximately in the middle (+7814 to +8038) and set C the region close to the 3′ end (+9978 to +10,217). Paxillin was enriched only at the region amplified by set A, suggesting that paxillin interacts with the very beginning of the enhancer sequence (Fig. 4B). Taken together, the results indicate that paxillin binds both key transcription elements – promoter and enhancer – and, therefore, might regulate their mutual interaction.

### Paxillin regulates the interaction between the *IGF2* P3 or *H19* promoter and the enhancer

**Fig. 5.**
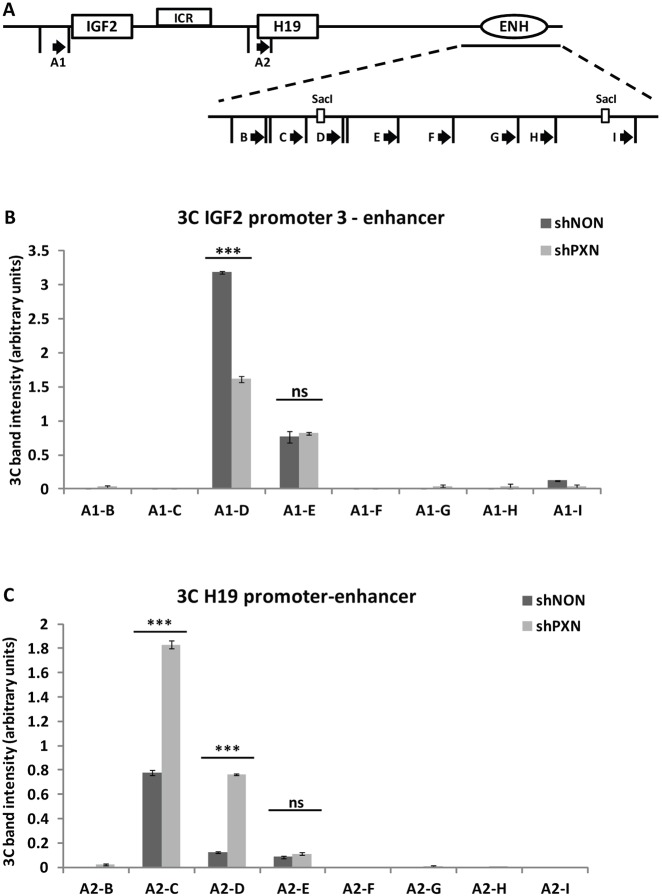
**Paxillin regulates the interactions between the *IGF2* P3 or *H19* promoter and the enhancer.** (A) Design of 3C analysis. Vertical lines show PstI restriction sites and black arrows represent the designed primers. (B) A chromatin conformation capture (3C) assay was performed using the *IGF2* P3 fragment (A1) as an anchor, and the interaction between the *IGF2* P3 fragment and fragments of enhancer (A1-B–A1-I) in HepG2 cells was assessed. The interaction frequency between *IGF2* P3 and the enhancer was significantly lower after paxillin knockdown (shPXN) compared to control (shNON). (C) A 3C assay was performed in the same way, but using the *H19* promoter fragment (A2) as an anchor examining the interaction between the *H19* promoter and the fragments of enhancer (A1-B–A1-I). Paxillin depletion increased the interaction frequency between *H19* promoter and the enhancer. The interaction frequency corresponds to the intensity of amplified PCR products; analyzed gels are shown in supplementary material Fig. S2. Data are shown as mean±s.d. (*n*≥3). ***P<0.001; ns, not significant (Student's *t*-test).

As we have shown that paxillin regulates the activation of both promoters through the shared enhancer and also occupies these regulatory elements, we speculated that paxillin might stimulate formation of long-range chromatin loops between the *IGF2* P3 or *H19* promoter and the enhancer. To test this hypothesis, we used chromatin conformation capture (3C) technology to study physical interactions between chromosomal regions (Dekker et al., 2002). Instead of mapping all the interactions at the *IGF2*/*H19* locus, we focused on comparing the interaction frequency between the *IGF2* P3 or *H19* promoter and the enhancer in control and paxillin-knockdown HepG2 cells. The interaction frequency was examined using the promoter as an anchor and the enhancer as a test region (schematically shown in Fig. 5A). The intensity of the amplified band corresponds to the interaction frequency between the tested regions (supplementary material Fig. S2). In agreement with the previous data, the interaction frequency between the *H19* promoter and the enhancer increased in the paxillin-knockdown cells (Fig. 5C). Correspondingly, the interaction frequency between the *IGF2* P3 and the enhancer was significantly lower after paxillin knockdown (Fig. 5B). In both cases, the interaction was detected at the 5′ end of the enhancer sequence. This result clearly confirms that paxillin does indeed regulate the activation of the *IGF2* P3 and *H19* promoters through the same enhancer, and that it does so in an opposite manner. In other words, paxillin promotes a contact of the *IGF2* P3 promoter with the enhancer while simultaneously blocking the activation of the *H19* promoter.

### Paxillin binds the cohesin proteins SMC1A and SMC3, and the mediator complex in the nucleus

**Fig. 6.**
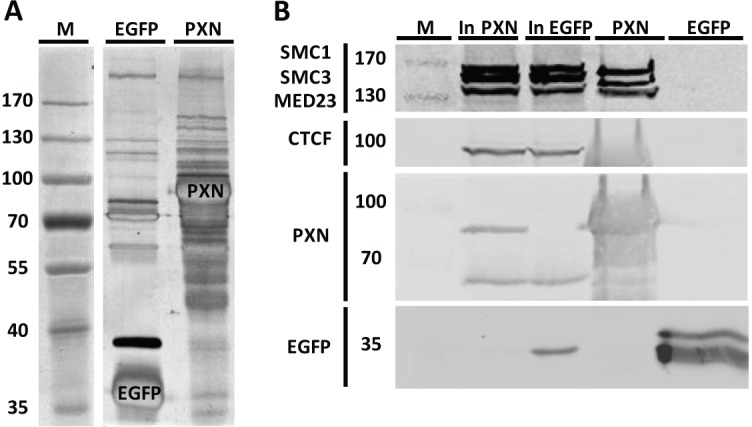
**Paxillin binds SMC1, SMC3 and MED23 in the nucleus.** (A) A representative gel of a paxillin pulldown stained with silver. HEK293 cells expressing only EGFP with an affinity tag were used as a control. (B) The specificity of the identified interactions was confirmed by western blotting. Cohesin proteins SMC1A (SMC1), SMC3 and MED23 were bound to PXN, but not to the control EGFP. No interaction of paxillin and CTCF was detected. Pulldown fractions from HEK293 cell stably expressing STrEP-EGFP–PXN (PXN) or STrEP-EGFP (EGFP used as a background control) were immunoblotted with the indicated antibodies. Anti-paxillin antibody (PXN) labels both endogenous paxillin (lower bands) as well as recombinant paxillin (upper band). In, input; M, molecular mass markers.

Finally, we searched for protein complexes involved in the regulatory action of paxillin exerted on the *IGF2*/*H19* promoters. We established a HEK293 cell line stably expressing EGFP–STrEP-paxillin (PXN) and identified proteins pulled down by the tagged protein by liquid chromatography tandem mass spectrometry (LC-MS-MS) (Fig. 6A). Proteins identified in the experiment are summarized in supplementary material Table S1. In addition to many known paxillin-binding proteins, our search also identified several new nuclear gene expression regulatory proteins that interacted with paxillin. Two proteins of cohesin family were repeatedly identified in paxillin pulldown experiments, namely the structural maintenance of chromosomes 1A protein (SMC1A) and the structural maintenance of chromosomes 3 protein (SMC3). Furthermore, we also identified three subunits of the mediator complex (MED), specifically MED15, MED24 and MED23. Although the number of peptides found was very low, no peptides were detected in the control indicating that the interaction is specific.

The specificity of the identified interactions was confirmed by western blotting (Fig. 6B). Both SMC1A and SMC3 proteins, as well as MED23 were found in the paxillin pulldown fraction, but not in the control, which is consistent with our mass spectrometry data. The low incidence of other mediator subunits in the pulldown prevented their identification on western blots. However, no CTCF was found in the pulldown fractions, showing that paxillin is not present in the cohesin–CTCF complexes. Thus, we present the evidence for the interaction between paxillin and gene expression regulatory proteins SMC1A, SMC3 and the mediator complex.

### Paxillin, cohesin and mediator co-occupy the *IGF2*/*H19* promoters and the enhancer

**Fig. 7.**
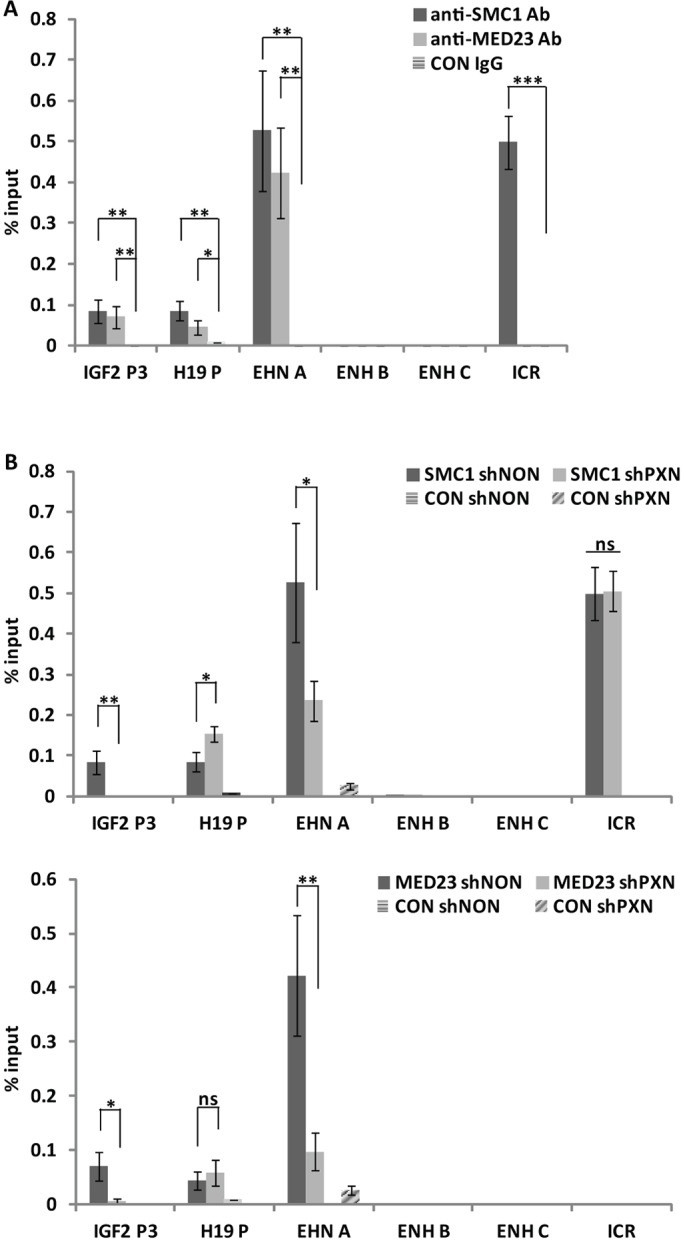
**Paxillin, cohesin and mediator co-occupy the *IGF2*/*H19* promoters and the enhancer.** (A) ChIP using anti-SMC1 or anti-MED23 antibody or rabbit IgG (CON IgG) showed specific enrichment of SMC1A (SMC1) and MED23 on the *IGF2* promoter, the *H19* promoter and the 5′ end of enhancer. See Fig. 4A for location of primer sets A, B and C. (B) Depletion of paxillin by shRNA (shPXN) significantly decreased the amount of SMC1 and MED23 on the enhancer compared to control (shNON). We detected less SMC1A and MED23 on the *IGF2* promoter (P3), and more SMC1A (but not MED23) on the *H19* promoter. Paxillin depletion did not change the binding of SMC1A to the ICR. Data are shown as mean±s.d. (*n*≥3). *P<0.05; **P<0.01; ***P<0.001; ns, *P*>0.05 (Student's *t*-test).

Next, we asked whether the cohesins and mediator complex bind to the same regions on the *IGF2*/*H19* promoters and the enhancer as paxillin. The cohesin subunit SMC1A and the mediator subunit 23 (MED23) were immunoprecipitated from crosslinked chromatin of HepG2 cells and their occupancy on the *H19–IGF2* regulatory elements tested. We found that both SMC1A and MED23 were enriched on the *IGF2* promoter, the *H19* promoter and specifically on the 5′ end of the enhancer, similar to paxillin (Fig. 7A). Finally, we examined whether knockdown of paxillin affected the binding of cohesin and mediator to these DNA elements. The depletion of paxillin by shRNA (shPXN) significantly decreased the amount of SMC1A and MED23 proteins on the enhancer (Fig. 7B). In accordance with our 3C data (see Fig. 5A–C), we detected less SMC1A and MED23 on the *IGF2* promoter (P3) (Fig. 7B), and more SMC1A (but not MED23) on the *H19* promoter (Fig. 7B). Paxillin depletion did not change the binding of SMC1A to the ICR (Fig. 7B) or the mRNA levels of SMC1A and MED23 (data not shown).

## DISCUSSION

Paxillin belongs to a class of focal adhesion proteins that shuttle between focal contacts and the nucleus. Its nuclear localization has been linked to the regulation of gene expression, especially that of the *H19* gene (Woods et al., 2002; Dong et al., 2009; Sen et al., 2012). Nevertheless, the mechanism of the gene cluster regulation as well as the *IGF2*/*H19* imprinting mechanism remain unknown.

In this paper we demonstrate, consistent with previous findings (Dong et al., 2009), that the depletion of paxillin upregulates *H19* gene expression in human HepG2 cells. Surprisingly, we did not detect a significant change in *IGF2* expression. This was unexpected because the *H19*–*IGF2* genes usually react reciprocally towards the regulating signals, including the depletion of CTCF or cohesin (Wendt et al., 2008; Nativio et al., 2009). Furthermore, depletion of these proteins also abrogates ICR methylation, thus disrupting monoallelic expression (Constancia et al., 2000; Wendt et al., 2008; Nativio et al., 2009). In the case of paxillin depletion, however, the ICR methylation was intact and the allelic expression of *H19* unaffected. Interestingly, whereas *H19* was transcribed from a single allele, *IGF2* was biallelicaly transcribed in both control and in paxillin-depleted cells. As the ICR insulation barrier responsible for *IGF2* imprinting was intact, there has to be another regulatory mechanism mediating this process, or alternatively, the imprinting mechanism for *IGF2* is dysfunctional. Indeed, reactivation of *IGF2* expression on the maternal allele has been previously reported in a number of human tumors and tumor cell lines (Li et al., 1995; Singer et al., 1995; Takeda et al., 1996; Zhang et al., 1997; Cui et al., 1998; Hofmann et al., 2002). However, *IGF2* has been also found to be expressed biallelically during normal development: in the choroid plexus and leptomeninges of both mouse (DeChiara et al., 1991) and man (Ohlsson et al., 1994), and postnatal human livers (Kalscheuer et al., 1993; Ohlsson et al., 1994; Davis, 1994). Thus, *IGF2* expression might be controlled by diverse regulatory mechanisms. Unfortunately, we could only examine the ICR methylation status and allele-specific expression in the SAOS2 cell line given that HepG2 cells are homozygous and, therefore, allelic expression cannot be distinguished by SNPs. However, we showed that depletion of paxillin in SAOS2 cells resulted in the same changes in *H19–IGF2* expression (supplementary material Fig. S3). The fact that similar changes occur in both cell lines after paxillin depletion suggests that the conclusions drawn from experiments using SAOS2 cells can also be applied to HepG2 cells.

Next, we showed that paxillin occupies promoters of *IGF2* and *H19* genes and their shared endodermal enhancer. Two different enhancer sequences for *IGF2*/*H19* have been identified so far, endodermal and mesodermal. Whereas the endodermal enhancer has been described in both mice and humans (Leighton et al., 1995b; Kopf et al., 1998; Ohana et al., 1999), the mesodermal one is more elusive and a putative mouse mesodermal enhancer region at 22–28 kb downstream of *H19* (Ishihara et al., 2000) has not been studied in human cells. In our experiments, we used the endodermal enhancer (+6033 to +10,972 bp relative to *H19*; delineated by SacI restriction sites). This sequence was originally identified in mice (Yoo-Warren et al., 1988; Arney, 2003) and later reported to stimulate the *H19* promoter in human endodermal cell lines such as HepG2 (Kopf et al., 1998; Ohana et al., 1999; Long and Spear, 2004; Varrault et al., 2006).

Using luciferase reporter plasmids, we showed that paxillin regulates the effect of the endodermal enhancer not only on the *H19* promoter but also on the *IGF2* promoter. As expected, the activity of the *H19* promoter increased after paxillin knockdown in accordance with the upregulation of *H19* expression in this genomic context. Interestingly, we observed decreased activity of the *IGF2* promoter. Thus, paxillin regulates both genes and has an opposite effect on the activity of the *IGF2* and *H19* promoters. Interestingly, nuclear accumulation of paxillin upon treatment with leptomycin B had a similar effect – downregulation of *H19* expression and upregulation of *IGF2* expression (supplementary material Fig. S4). This suggests that it is not the total levels but rather the nuclear levels of paxillin that are more important for its nuclear function. As the expression of the *IGF2*/*H19* genes is determined not only by paxillin, but also by cohesins, CTCF, vigilin and ZAC1 (Varrault et al., 2006; Wendt et al., 2008; Nativio et al., 2009; Liu et al., 2014), the ratio of the regulatory proteins is apparently crucial for the resultant expression of the *IGF2* and *H19* genes. Thus, elevated levels of paxillin in the nucleus (regardless of the total levels of paxillin) can prevail over other regulatory pathways. Further examination of the proteins regulating the *IGF2*/*H19* gene cluster and especially their mutual crosstalk should bring more insight on the mechanism.

**Fig. 8.**
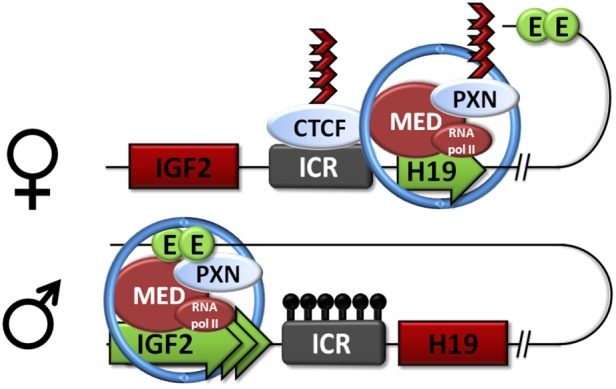
**Model of *IGF2*/*H19* regulation by paxillin.** Paxillin (PXN) assembles with cohesin and mediator (MED), and engages in the mediation of the long-range chromosomal interactions between the *IGF2* or *H19* promoter and the shared distal enhancer (E) regulating their transcription. Specifically, paxillin enhances the interaction between the enhancer and the *IGF2* promoter, but blocks the *H19*-promoter–enhancer interaction, resulting in stimulation of *IGF2* and suppression of *H19* expression. The model shows the paternal and maternal allele of the genes. Red box, inactive gene; green box, active gene; black lollipops, methylation; blue circle, cohesin complex; dark red arrows, ICR barrier.

We then demonstrated that paxillin regulates the formation of long-range interactions between the promoters of the *H19* and *IGF2* genes and their shared enhancer. Interestingly, we identified an interaction of paxillin with the cohesin complex (subunits SMC1A and SMC3), which has been established as the main regulator of long-range chromatin interactions at the *IGF2*/*H19* cluster (Wendt et al., 2008; Nativio et al., 2009). Moreover, cohesin has been found to colocalize with the mediator complex (Kagey et al., 2010). These proteins have been shown to mediate long-range interactions between enhancers and promoters of key pluripotency transcription factors in mouse embryonic stem cells (Conaway and Conaway, 2011). In this process, the ring-shaped structure of cohesin is employed to lock together the enhancer and the promoter regions of a single chromatid once they are brought into proximity by their simultaneous binding to the activator mediator (Hadjur et al., 2009). We found cohesin, as well as subunits of mediator, specifically its tail module (Malik and Roeder, 2010), among the paxillin-associated proteins in pulldown experiments. Furthermore, cohesin and mediator bound to the *IGF2*/*H19* promoters and the 5′ end of the enhancer, where paxillin is also enriched, and importantly, the depletion of paxillin changed their amount on these DNA elements. These data suggest that SMC1A and MED23 play a role in paxillin-dependent regulation of the *H19*–*IGF2* gene cluster. Here, we propose a model (Fig. 8) where paxillin assembles with cohesin and mediator, and mediates the long-range chromosomal interactions between *IGF2* or *H19* promoter and the shared distal enhancer, thus regulating their transcription. Specifically, paxillin enhances the interaction between the enhancer and the *IGF2* promoter, but blocks the *H19* promoter–enhancer interaction, resulting in stimulation of *IGF2* and suppression of *H19* expression. Our model also explains the significant decrease in cell proliferation after paxillin depletion, as it causes upregulation of *H19* non-coding RNA, thus giving rise to proliferation-repressing miRNAs (Gabory et al., 2010). Both *IGF2* and *H19* genes are widely expressed during embryonic development, after which they are downregulated (except in skeletal muscle; Brunkow and Tilghman, 1991; Delaval and Feil, 2004). Their proper expression is therefore crucial especially during fetal development (Delaval and Feil, 2004; Pannetier and Feil, 2007; Gabory et al., 2010), and even though their regulatory mechanisms are preserved in cultured somatic cells, we assume that the key significance of the regulatory role of paxillin lies in development.

Based on our findings, we propose a new regulatory mechanism for the *IGF2*/*H19* locus employing paxillin in a complex with cohesin and mediator; this complex mediates promoter–enhancer interactions. Similar interactions have also been observed in androgen-receptor-dependent genes (Wang et al., 2005). Considering that the androgen-receptor–paxillin complex occupies promoters of androgen-receptor-responsive genes, such as PSA (also known as KLK3) or NKX3-1 in prostate cancer cell line (Sen et al., 2012), it is worth exploring whether paxillin is employed here in a similar way. Given the fact that paxillin is a focal adhesion protein, this shows that cells might regulate gene expression by taking advantage of the physical barrier formed by the nuclear envelope and thus controlling access of regulatory factors to their target genes.

## MATERIALS AND METHODS

### Cell lines

SAOS2 and HEK293 cells were grown in Dulbecco's modified Eagle's medium (DMEM), HepG2 cells in RPMI-1640 medium, both of them supplemented with 10% fetal bovine serum (FBS) and antibiotics. Cells were incubated at 37°C in a humidified 5% CO_2_ and air atmosphere. Plasmid transfections were performed using Lipofectamine 2000 (Invitrogen, Carlsbad, CA) according to manufacturer's protocol. Stable cell lines were established by selection with geneticin G-418 (800 µg/ml, Gibco, Life Technologies, Carlsbad, CA) or puromycin (2 µg/ml, Gibco, Life Technologies, Carlsbad, CA).

### Antibodies

The following antibodies were used in the present study: rabbit monoclonal against the N-terminal of paxillin (Merck Millipore, Billerica, MA); rabbit polyclonal anti-DRIP130 antibody (MED23, Abcam, Cambridge, UK); rabbit polyclonal anti-SMC1A antibody (ChIP Grade, Abcam); goat polyclonal anti-SMC3 antibody (Santa Cruz Biotechnology, Inc., Dallas, TX); rabbit polyclonal anti-CTCF antibody (ChIP Grade, Abcam); rabbit polyclonal anti-H3K4me2 antibody (Merck Millipore); rabbit polyclonal anti-GFP (Molecular Probes, Life Technologies, Carlsbad, CA); mouse monoclonal anti-actin antibody (Sigma-Aldrich, St Louis, MO); and mouse monoclonal anti-BrdU antibody (Sigma-Aldrich).

### Cloning

The pSTrEP-GFP-PXN vector was prepared by ligation of full-length human paxillin cDNA (U14588.1; a generous gift from Ravi Salgia, The University of Chicago School of Medicine, Chicago, IL) into pSTrEP-EGFP-C3 previously prepared by insertion of the One-StrEP-tag sequence (IBA GmbH, Göttingen, Germany) into pEGFP-C3 (Dzijak et al., 2012). Firefly luciferase reporter vectors p*H19*P-luc and *IGF2*P3-luc were constructed by inserting the *H19* promoter (−819 to +13 bp relative to *H19* transcription start site) or *IGF2* promoter 3 (−499 to +13 bp relative to *IGF2* transcript 3 transcription start site; +7858 to +8369 bp relative to the *IGF2* exon 1 start) into pGL4.10 (Promega, Madison, WI) using the KpnI and HindIII sites. The *H19* endodermal enhancer sequence (+6033 to +10,972 bp) was inserted into the SalI site of p*H19*P-luc or p*IGF2*P3-luc to create p*H19*P-luc-E or p*IGF2*P3-luc-E, respectively.

### Lentiviral transduction

The lentiviral particles containing shRNAs were generated in HEK293T cells using the Addgene (Cambridge, MA) two-plasmid system (pMD2G, psPAX2) and pLKO.1 vectors containing anti-paxillin shRNA (shPXN) or non-targeting shRNA (shNON; Non-Target shRNA Control, Sigma-Aldrich), according to the manufacturer's protocol. Five clones of shPXN were purchased and screened for knockdown efficiency and the TRCN0000123136 clone was selected for further experiments. The viral supernatants were spun down, concentrated by PEG precipitation (PEG 6000, Sigma-Aldrich), aliquoted and stored at −80°C. HepG2 or SAOS2 cells were seeded on a 24-well plate 24 h before transduction. Virus-containing supernatant was added to the medium, incubated overnight and, after 24 h, replaced with fresh medium containing puromycin. Protein, RNA or DNA content was analyzed at 5 days post transduction unless otherwise stated.

### BrdU incorporation assay

Cells were seeded on glass coverslips overnight, BrdU (Sigma-Aldrich) was added to the medium (final concentration 1 µM) and cells were cultured for 1 h. Subsequently, the cells were fixed in 3% paraformaldehyde, and DNA was denatured by incubation in 2 M HCl for 30 min and immediately neutralized in 0.1 M borate buffer, pH 9.0. After permeabilization with 0.1% Triton X-100 (w/v) and several washes in PBS, cells were incubated with primary anti-BrdU antibody for 1 h, followed by anti-rabbit-IgG secondary antibody for 30 min and mounted with Mowiol (Sigma-Aldrich) containing 0.1 μg/ml DAPI (Sigma-Aldrich). Images were acquired with a confocal microscope (Leica TCS SP) and ratios of BrdU-positive to all cells were obtained upon counting the cells using ImageJ.

### Western blotting

Cells were homogenized in SDS lysis buffer (60 mM Tris-HCl pH 6.80, 10% glycerol, 2% SDS) and briefly sonicated. The lysates were cleared by centrifugation and total protein content was measured by using a Bradford assay. Equal amounts of proteins were loaded onto 10% polyacrylamide gel, proteins separated by SDS-PAGE and transferred onto a nitrocellulose membrane. The blots were incubated with appropriate primary and fluorescently conjugated secondary antibodies and scanned on a Li-cor Odyssey imager (Lincoln, NE).

### Mass spectrometry analysis

Samples were prepared as follows: proteins were eluted with 50 mM Tris-HCl pH 8.00, 2.5 mM desthiobiotin, and eluates digested with 1 µg of trypsin (Trypsin Gold, Promega, Madison, WI) overnight at 30°C. Peptides were desalted using a microtrap column (MichromBioresources, CA), dried down and dissolved for LC-MS analysis performed as previously described (Stodůlková et al., 2008), with minor modifications. The mass spectrometric analysis (MALDI-TOF-TOF type) was performed using the Mascot 2.0 search engine (Matrix Science, Boston, MA) with the following search parameters: SwissProt database, taxonomy human, trypsin specificity, no fixed modifications, oxidized methionine as a variable modification, MS-tolerance of 50 ppm and MS/MS tolerance 0.5 Da. Only proteins identified on three peptides with Mascot score above 30 were considered to be positive hits.

### RNA isolation and qPCR

Total RNA was isolated using GenElute Miniprep Kit (Sigma-Aldrich) according to the manufacturer's protocol. RNA was treated with RNase-free DNase I for 30 min at room temperature. The concentration of RNA was measured by spectrophotometry and RNA integrity was checked on a denaturing agarose gel. A total of 100 ng of RNA was reverse-transcribed with random hexamer primers using TaqMan Reverse Transcription Reagents (Applied Biosystems, Life Technologies, Carlsbad, CA). Quantitative real-time PCR (qPCR) was performed on ABI Prism 7300 instrument using SYBR Green PCR Master Mix (Applied Biosystems, Life Technologies) and appropriate primers. Data were evaluated with ΔΔCt method and transcript levels normalized to those of the GAPDH gene. Primers used for RT-PCR are shown in supplementary material Table S2.

### Pulldown assays

Cells were harvested by trypsinization, spun down, washed twice in ice-cold PBS and resuspended in a lysis buffer [20 mM HEPES-KOH pH 7.40, 5 mM CH_3_COOK, 150 mM NaCl, 0.5 mM MgCl_2_, 0.5 mM DTT and 0.5% (w/v) Triton X-100] containing EDTA-free protease inhibitor cocktail (cOmplete, Roche, Basel, Switzerland) and phosphatase inhibitor cocktail (Phos STOP, Roche). The lysate was centrifuged at 16,000 ***g*** for 20 min to pellet insoluble proteins. STrEP-T-actin-coupled sepharose beads (IBA GMBH, Göttingen, Germany) were washed several times with the lysis buffer, added to the lysate and the mixture incubated for 2 h. The beads were then spun down, washed five times for 10 min with the lysis buffer and finally the bound proteins were eluted in a native form using lysis buffer or 50 mM Tris-HCl pH 8.00 (for MS analysis) containing 2.5 mM desthiobiotin. The ‘pulldown’ eluate was analyzed by SDS-PAGE or MS and proteins visualized by staining the polyacrylamide gel with SilverQuest kit according to manufacturer's protocol (Invitrogen, Carlsbad, CA). The eluates were concentrated by ultrafiltration (Ultracel 10K, Merck Millipore, Billerica, MA).

### Genotyping and allele-specific expression assay

The region covering *IGF2* SNP rs680 and *H19* rs2839704 was amplified from SAOS2 genomic DNA with appropriate primers (see supplementary material Table S2) and sequenced. RNA isolated from SAOS2 shNON or SAOS2 shPXN cells was reverse transcribed, amplified with the same primers and digested with RsaI or ApaI for *H19* SNP or *IGF2* SNP, respectively. Fragments were resolved on 3% agarose gel. Absence of DNA contamination in the RNA samples was checked by parallel controls without reverse transcription.

### Methylation analysis

Genomic DNA isolated from SAOS2 cells was subjected to bisulfite conversion using the Methylamp DNA Modification Kit (Epigentek, Farmingdale, NY) according to the manufacturer's protocol. Bisulfite treatment efficiently converts unmethylated cytosine into uracil, whereas 5-methylcytosine remains unchanged (Hayatsu et al., 1970). Treated DNA was purified and amplified by PCR, with primers corresponding to the ICR region. Primers specific for bisulfite-treated DNA were designed with the Bisulfite Primer Seeker program (Zymo Research, Irvine, CA; see supplementary material Table S2). The amplified PCR product was inserted into pJET vector and transformed into competent cells. Plasmid DNA isolated from at least 30 bacterial colonies (for each shNON and shPXN variant) was sequenced.

### Chromatin immunoprecipitation

ChIP was performed using MAGnify Chromatin Immunoprecipitation System (Invitrogen) according to the manufacturer's protocol. Around 300,000 cells were used as a starting material, and DNA was sheared with 15 sonication cycles (30 s ON, 30 s OFF; intensity HIGH) using a Bioruptor Next Gen sonicator (Diagenode, Seraing, Belgium). Antibodies against paxillin, SMC1, MED23, CTCF, control IgG (negative control) or H3K4me2 (positive control) were used. Input and immunoprecipitated DNA levels were quantified by qPCR. Enrichment was calculated as the amount in the immunoprecipitation over input and further normalized to a region where paxillin does not bind (GAPDH-coding region). ChIP primers are shown in supplementary material Table S2.

### Luciferase reporter assay

The preparation of Firefly luciferase reporter vectors p*H19*P-luc, p*IGF2*P3-luc, p*H19*-luc-E, p*IGF2*P3-luc-E (see ‘Cloning’ for details) was adopted from Ishihara et al., 2006. These constructs were co-transfected with *Renilla* luciferase reporter vector pGL4.74 into shPXN or shNON HepG2 cells at 4 days after lentiviral transduction. After 24 h, all cells were lysed and luciferase activity of both species measured according to Dual-Glo^®^Luciferase Assay System protocol (Promega, Madison, WI), using Modulus™ II Microplate Multimode Reader (Turner Biosystems, Sunnyvale, CA). Data were normalized to *Renilla* luciferase activity and all assays performed in triplicate and repeated at least three times.

### Chromatin conformation capture assay

The technique was adopted from Hagège et al. (Hagège et al., 2007), except for the final semiquantitative analyses (Naumova et al., 2012). Briefly, cells expressing shNON or shPXN (5 days after transduction) were crosslinked with 1% formaldehyde, lysed and digested with PstI restriction enzyme. After ligation, samples were reverse-crosslinked overnight at 65°C. Isolated DNA was amplified with corresponding primers (schematically shown in Fig. 5A; see supplementary material Table S1 for the sequences) and PCR products resolved on 3% agarose gels. The amount of DNA input was first titrated and bands analyzed semi-quantitatively using ImageJ software; the background was subtracted and data normalized to a total DNA input unaffected by the restriction digest (ICR region). Three biological replicates were prepared and analyzed in three technical repeats.

## Supplementary Material

Supplementary Material
