# Supplementary material for: Paxillin-dependent regulation of IGF2 and H19 gene cluster expression
Source: J Cell Sci. 2015 Aug 15;128(16):3106–16. doi: 10.1242/jcs.170985 (PMC4541046; doi:10.1242/jcs.170985)
Supplement: Supplementary Material [file supp_128_16_3106__index.html]

Supplementary Material 

# Paxillin-dependent regulation of *IGF2* and *H19* gene cluster expression

## JCS170985 Supplementary Material

- Supplementary Material
