# Supplementary material for: Paxillin-dependent regulation of IGF2 and H19 gene cluster expression
Source: J Cell Sci. 2015 Aug 15;128(16):3106–16. doi: 10.1242/jcs.170985 (PMC4541046; doi:10.1242/jcs.170985)
Supplement: Supplementary Material [file supp_jcs.170985_JCS170985supp.pdf]

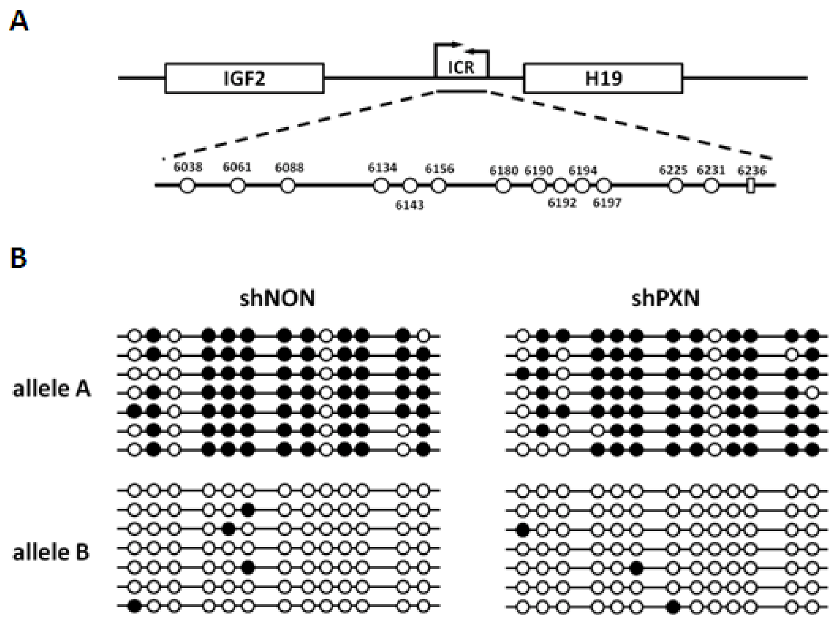

Fig. S1 Methylation of ICR is preserved in paxillin-depleted cells

A) A diagram of the analyzed ICR segment. The enlargement shows specific CpG islands. The rectangle represents single nucleotide polymorphism employed to distinguish between the alleles, arrows show positions of primers employed in amplicifation; base numbering according to GenBank accession number AF087017. **B)** Sequencing of bisulphite-treated genomic DNA from paxillin-depleted (shPXN) and control (shNON) SAOS2 cells demonstrated preserved methylation of one allele. The alleles were distinguished by SNP rs2071094. Each line represents a single sequenced PCR product; black/white circles – methylated/unmethylated CpGs.

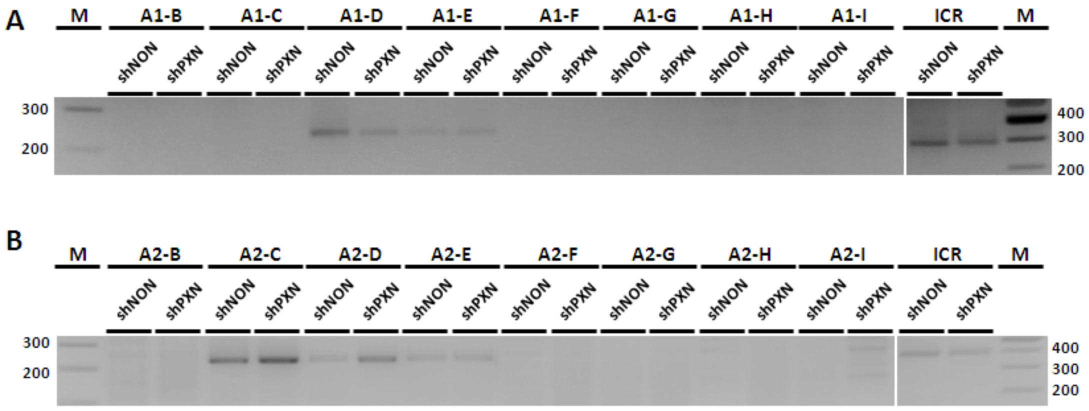

Fig. S2 Gels showing amplified products of Chromatin conformation capture assay (3C)

Chromatin conformation capture assay (3C) was performed as outlined in Fig. 5A and in the corresponding chapter in Results. **A)** The amplified PCR bands using the *IGF2* P3 fragment (primer A1) and the enhancer fragments (primers B-I) were resolved on a 3% agarose gel and semiquantitatively analysed. **B)** The amplified PCR bands using the *H19* fragment (primer A2) and the enhancer fragments (primers B-I) were resolved on a 3% agarose gel and semiquantitatively analysed. In both cases the background was subtracted and data normalized to the loading control (ICR region, 400 bp, unaffected by digestion). The primers were designed to amplify fragments of 200 - 230 bp long.

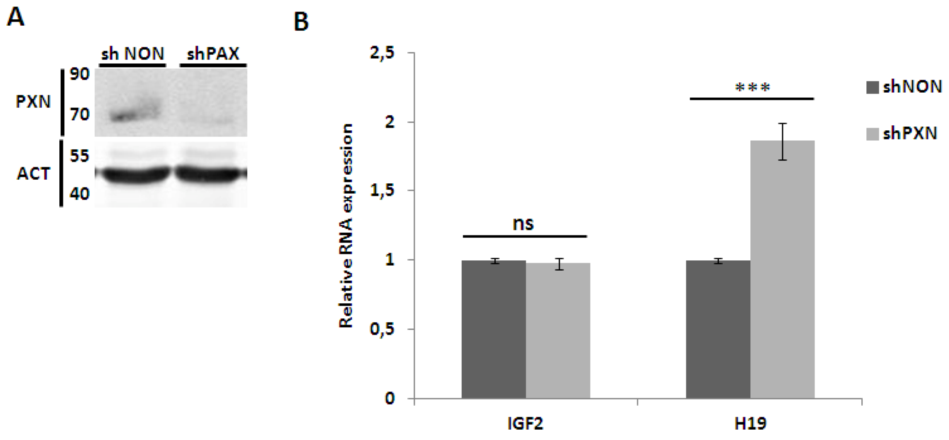

Fig. S3 Knockdown of paxillin in SAOS2 cells results in similar expression changes of *IGF2* and *H19* genes

A) Protein level of paxillin in paxillin-depleted (shPXN) and control (shNON) SAOS2 cells. Actin (ACT) was used as a control of protein amount. **B)** QPCR analysis showed that paxillin depletion (shPXN) in SAOS2 cells results in the upregulation of *H19* compared to control (shNON), but no effect on *IGF2* was observed. Similar results were obtained when HepG2 cells were used. Data were normalized to the *GAPDH* gene and then to the control shNON sample. Data are shown as means ± s.d.

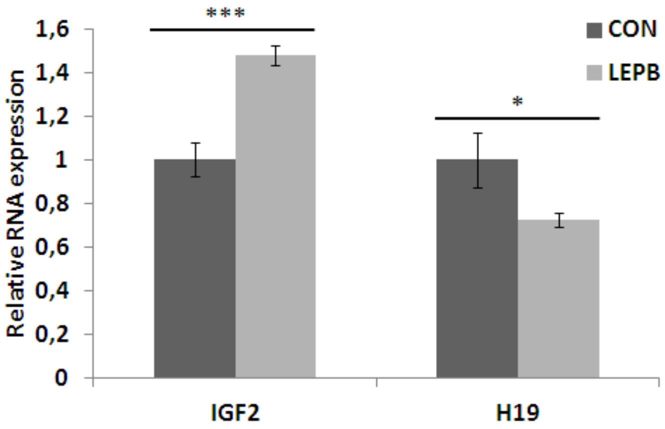

Fig. S4 Accumulation of paxillin in the nuclei of HepG2 cells affects the expression of *IGF2* and *H19* genes

Accumulation of paxillin in the nuclei of HepG2 cells upon treatment with leptomycin B (10 ng/ml, 2h) causes upregulation of *IGF2* and dowregulation of *H19* gene. Data were normalized to the *GAPDH* gene and then to the control (CON) sample. Data are shown as means ± s.d.; ***p < 0.001; *p< 0.05; Student`s t-test.

Table S1. Proteins identified by mass spectrometry in paxillin pull-down.

Protein	Symbol	MW (kDa)	Peptides	SC (%)
**Structural maintenance of chromosomes 1A**	SMC1A	141	13	10
**Structural maintenance of chromosomes 3**	SMC3	143	12	9
**Mediator of RNA polymerase II subunit 15**	MED15	86	1	2
**Mediator of RNA polymerase II subunit 23**	MED23	156	1	2
**Mediator of RNA polymerase II subunit 24**	MED24	110	1	2
**Focal adhesion kinase 1**	FAK1	120	22	28
**ARF GTP-ase activating protein 1**	GIT1	84	39	51
**ARF GTP-ase activating protein 2**	GIT2	85	24	38
**Serine/threonine protein kinase 1**	PAK1	60	18	38
**Serine/threonine protein kinase 2**	PAK2	58	13	34
**Rho guanine nucleotide exchange factor 7**	ARHGEF7	90	31	31
**Rho guanine nucleotide exchange factor 6**	ARHGEF6	87	15	18
**Tubulin alpha**	TUBA	50	9	29
**Tubulin beta**	TUBB	50	18	44

Table S2. Primers used in this study.

**qPCR**	
QPXN_FW	AAAGTTGCGGGGCATAGAC
QPXN_REV	AAGAACACAGGCCGTTTGGA
QGAPDH_FW	AAGGTGAAGGTCGGAGTCAA
QGAPDH_REV	AATGAAGGGTCATTGATG
QIGF2_FW	GTGGCATCGTTGAGGAGTG
QIGF2_REV	CACGTCCCTCTCGGACTTG
QH19_FW	CTTTACAACCACTGCACTACCTGAC
QH19_REV	GATGGTGTCTTTGATGTTGGGCTGA
QSMC1A_FW	CATCAAAGCTCGTAACTTCCTCG
QSMC1A_REV	CCCCAGAACGACTAATCTCTTCA
QMED23_FW	TGCAGTCACTGAGATCAGGAA
QMED23_REV	CTACAGCGACCACAAATGGAG
**SNPs genotyping, allele-specific expression**	
SNP_IGF2_FW	CAACAACCCCTCTAAAACTAATTGGC
SNP_IGF2_REV	CCTCCTTTGGTCTTACTGGG
SNP_H19_FW	TACAACCACTGCACTACCTG
SNP_H19_REV	TGGAATGCTTGAAGGCTGCT
**Bisulphite treatment**	
ICR_BT_5962 FW	TGTTGAAGGTTGGGGAGATGGGA
ICR_BT_6413 REV	CCCAAACCATAACACTAAAACCCTC
**Chromatin immunoprecipitation**	
ChIP_GAPDH_FW	ACATCAAGAAGGTGGTGAAG
ChIP_GAPDH_REV	AGCTTGACAAAGTGGTCGTTG
ChIP_ENH A_FW	CCCAGGAAGATAAATGATTTCCTCCTC
ChIP_ENH A_REV	TGGGTCTCAGGGAATGGTCTC
ChIP_ ENH B_FW	CAAAGACATTTAGAAAAACCGGTTTAG
ChIP_ ENH B_REV	TGCAGACATCACTGTTGACACAC
ChIP_ ENH C_FW	ATGGGGGAGATGGACAACAG
ChIP_ ENH C_REV	GGGGGTCCATTTCTAGGCTCT
ChIP_ IGF2 P3_FW	AAATTTTGGGGAACGCAAGG
ChIP_ IGF2 P3_REV	CCCAAACCTGTAATCTATTTTTCTGGA
ChIP_ CTCF_FW	CTCCTTCGGTCTCACCGCCTGGAT
ChIP_ CTCF_REV	CCTTAGACGGAGTCGGAGCTG
**Chromatin Conformation Capture (3C)**	
3C_A1	AGAGCGGGAAGACAGGCAGT
3C_A2	GAAAACATCAACACAGCCAGGG
3C_B	ACAGGCCAATTTGACTTACCCAAG
3C_C	ATCTCCCAGCACTGCCCAT
3C_D	CTCTACCCCGCTCCCTGGC
3C_E	CAACACCTGGCTGGACACAG
3C_F	CACGTTGCAGAAAACTGGCTG
3C_G	AGCACAGAGAGGCACACGTAGG
3C_H	GTTCACAGGAAGCATTCGAGATAAG
3C_I	GAGACCCCATCTCTACCAAATATCA
**IGF2 promoter-specific expression**	
IGF2_T1_FW	GCCCCAACTGCGAGGCAGAG
IGF2_T2_FW	CGTAGAGCAACTCGGATTTGG
IGF2_T3_FW	CCAGAGCGGCGCTGGCAG
IGF2_T4_FW	TTCCTCCTCCTCCTCCTGCCCCAG
IGF2_T_REV	CTGAAGTAGAAGCCGCGG
